# Kinome-wide CRISPR-Cas9 knockout screens revealed *PLK1* as a therapeutic target for osteosarcoma

**DOI:** 10.1038/s41420-023-01526-7

**Published:** 2023-07-07

**Authors:** Renxian Wang, Dingding Wang, Xueshan Bai, Jianxun Guo, Songxia Xia, Yuning Cheng, Yani Gu, Qian Wang, Jingjun Nie, Dafu Chen, Weifeng Liu, Junbo Liang

**Affiliations:** 1 Laboratory of Bone Tissue Engineering, Beijing Laboratory of Biomedical Materials, National Center for Orthopaedics, Beijing Research Institute of Traumatology and Orthopaedics, Beijing Jishuitan Hospital, Capital Medical University, Beijing, China; 2 State Key Laboratory of Medical Molecular Biology, Department of Biochemistry and Molecular Biology, Institute of Basic Medical Sciences Chinese Academy of Medical Sciences, School of Basic Medicine Peking Union Medical College, Beijing, China; 3 Cranio-Maxillo-Facial Surgery Department, Plastic Surgery Hospital, Chinese Academy of Medical Sciences & Peking Union Medical College, Beijing, China; 4 Department of Orthopaedic Oncology Surgery, Beijing Jishuitan Hospital, Capital Medical University, Beijing, China

**Keywords:** Bone cancer, Target validation, Functional genomics, Mitosis

## Abstract

Osteosarcoma is the most common malignant bone tumor, tending to be aggressive and recurrent. The therapeutic development for treating osteosarcoma has been largely hampered by the lack of effective and specific targets. Using kinome-wide CRISPR-Cas9 knockout screens, we systematically revealed a cohort of kinases essential for the survival and growth of human osteosarcoma cells, in which Polo-like kinase 1 (*PLK1*) appeared as a specific prominent hit. *PLK1* knockout substantially inhibited proliferation of osteosarcoma cells in vitro and the tumor growth of osteosarcoma xenograft in vivo. Volasertib, a potent experimental PLK1 inhibitor, can effectively inhibit the growth of the osteosarcoma cell lines in vitro. It can also disrupt the development of tumors in the patient-derived xenograft (PDX) models in vivo. Furthermore, we confirmed that the mode of action (MoA) of volasertib is primarily mediated by the cell-cycle arrest and apoptosis triggered by DNA damage. As PLK1 inhibitors are entering phase III clinical trials, our findings provide important insights into the efficacy and MoA of the relevant therapeutic approach for combating osteosarcoma.

## Introduction

Osteosarcoma is the most common malignant bone tumor with generally poor prognosis in children and adolescents [1–5]. The standard therapeutic strategy based on the combination of chemotherapy and surgery is struggling with delivering optimal outcomes for the patients. Recent development of immunotherapy and targeted therapies are creating exciting opportunities for treating various cancer. However, the most available therapeutic options including PD-1/PD-L1 inhibitors and multi-tyrosine kinase inhibitors (mTKIs) are less effective on osteosarcoma [6–9].Thus, there is a pressing need in developing new strategies for alternative therapies as well as to improve the efficacies of current therapeutic treatments.

CRISPR-Cas9 technology enables functional genomics approaches to identify new targets and unravel novel underlying mechanisms for combating various medical conditions and diseases [10, 11]. As the most abundantly annotated post-translational modifications (PTMs), protein phosphorylation plays critical roles in wide spectrums of physiological and pathological conditions including cancer [12]. Kinase inhibitors, the largest class of tumor drug targets, account for over a quarter of projects under development [13]. For example, Mobocertinib, the EGFR tyrosine kinase inhibitor, received accelerated FDA approval in 2021 for the treatment of patients with EGFRex20ins-mutant NSCLC [14].

By exploring the human kinome, we discovered a cohort of genes required for human osteosarcoma cells growth and survival, in which *PLK1* was the top hit. More importantly, we confirmed that Volasertib, a frontline clinical candidate of PLK1 inhibitor, is effective in suppressing the growth of multiple osteosarcoma cell lines in vitro as well as the development of tumors in the patient-derived xenograft (PDX) models in vivo by disrupting the cell cycle and triggering apoptosis.

## Results

### Identification of the kinases essential for human osteosarcoma cells by the kinome-wide CRISPR-Cas9 screen

**Fig. 1:**
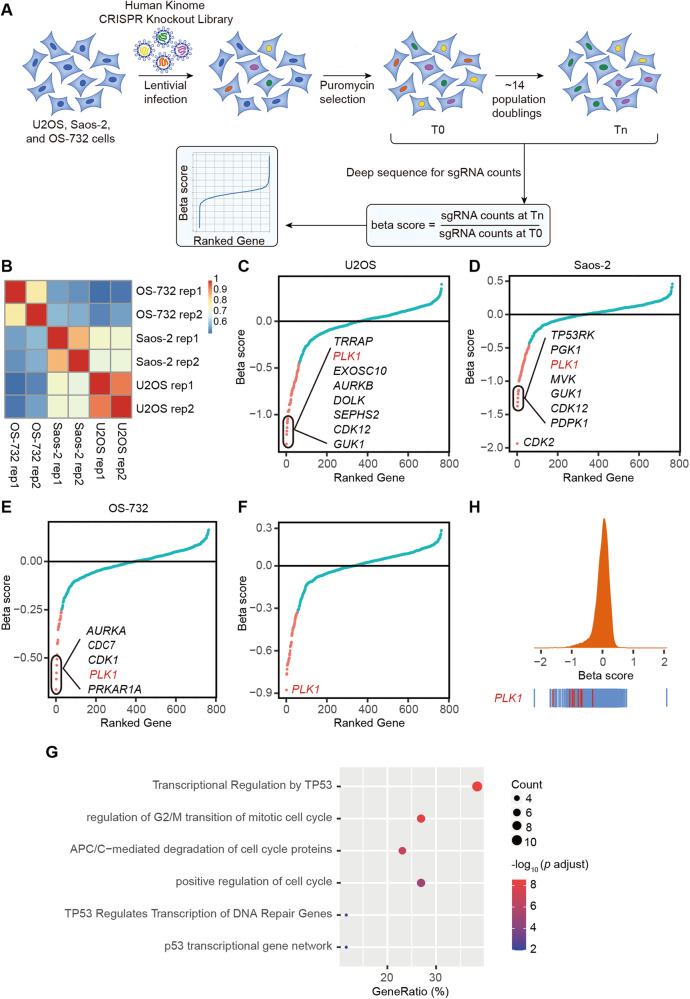
Kinome-wide CRISPR-Cas9 knockout screens reveal a cohort of kinases essential for human osteosarcoma cells. **A** Workflow of CRISPR-Cas9 knockout screen using human kinome CRISPR knockout library (Brunello) in U2OS, Saos-2 and OS-732 osteosarcoma cancer cell lines. Two infection replicates for each cell line was carried out. **B** Heatmap of Pearson correlation coefficients of gene beta scores across cell lines as well as replicates. Beta score was calculated using MAGeCK MLE algorithm. Top essential genes in knockout screen of U2OS (**C**), Saos-2 (**D**), and OS-732 cells (**E**). **F** Top essential genes of osteosarcoma, which used U2OS, Saos-2 and OS-732 cells as biological replicates. **G** Pathway enrichment of the essential genes using Metascape database. **H** Frequency histograms of sgRNA beta scores showing essentiality of *PLK1* in three osteosarcoma cells as biological replicates. Lines representing the beta scores of individual sgRNAs targeting *PLK1* are marked red.

To systematically explore the opportunities in targeting protein kinases, we screened three independent human osteosarcoma cancer cell lines, U2OS, Saos-2 and OS-732 using human kinome-wide CRISPR-Cas9 knockout library (Brunello) [15]. This allows us to individually target 763 human kinases with a collection of 6,104 unique sgRNAs and 100 non-targeting sgRNAs, for which a low multiplicity of infection (MOI, ~0.3) was established to ensure the specificity in the sgRNA-mediated loss of function (Fig. 1A). Using MAGeCK MLE algorithm [16], we calculated the beta score of genes from two independent replicates by comparing the sgRNA abundance between Tn and T0 populations (Fig. 1A). Pearson correlation coefficients (Fig. 1B; Supplementary Fig. S1A–C) confirmed high correlations across cell lines (0.51–0.74) as well as replicates (0.79–0.93). We first identified the essential genes for each osteosarcoma cell line (Fig. 1C–E, and Table S1). Using three different cell lines as biological replicates, we further evaluated the kinases essential for human osteosarcoma cells, revealing 26 significant candidate hits (beta score < −0.5 and false discovery rate (FDR) < 1%) (Fig. 1F and Table S1), which were enriched in pathways associated with cell cycle and TP53-regulated transcription (Fig. 1G).

Next, we used three inhibitors (Dinaciclib, Alisertib, or Barasertib), which targeted the top candidate hits (CDK members, AURKA, or AURKB) to validate the screen results in multiple osteosarcoma cell lines. Cell growth was significantly suppressed by targeting these identified gene respectively with the designated inhibitor (Supplementary Fig. S2A–C), thereby validating the screen. Importantly, *PLK1*, ranked as the top hit in our screen (Fig. 1F, H; Supplementary Fig. S3A–C), is under active pharmaceutical development (www.clinicaltrials.gov).

### *PLK1* expression is dysregulated in osteosarcoma and sarcoma, and is associated with poor prognosis

**Fig. 2:**
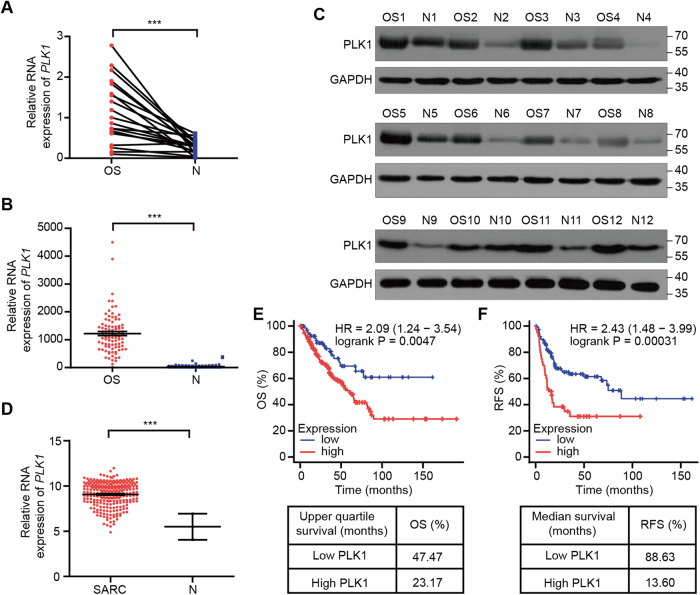
*PLK1* is over-expressed in osteosarcoma and sarcoma, and higher *PLK1* expression is associated with poor prognosis. **A** qRT-PCR analysis displaying relative *PLK1* mRNA expression in osteosarcoma and matched adjacent normal tissue from 20 osteosarcoma patients. **B** TNMplot showing expression of *PLK1* in osteosarcoma samples (*n* = 88) and non-cancerous samples (*N* = 564) in TARGET database. **C** Western blot analysis of PLK1 expression in osteosarcoma and matched normal tissues from 12 osteosarcoma patients. **D** Relative RNA expression of *PLK1* in sarcoma and non-cancerous samples in TCGA database analyzed by UCSC Xena web tool. Kaplan-Meier Plotter analysis of OS (**E**) and RFS (**F**) of sarcoma patients with high and low *PLK1* expression in the TCGA dataset. Values indicate mean ± SEM and unpaired and two-tailed t-tests were used to determine *P* values. *p < 0.05; **p < 0.01; ***p < 0.001.

In order to evaluate the clinical significance, we first analyzed *PLK1* expression in osteosarcoma. qRT-PCR analysis revealed that *PLK1* mRNA levels were higher in clinical osteosarcoma tissues (*n* = 20, 18/20 primary, 1/20 lung metastases, and 1/20 case breast metastases) than that in adjacent normal tissues (Fig. 2A), in line with the result by analyzing the data from osteosarcoma patients in the TARGET database (Fig. 2B). Additionally, PLK1 protein levels were also upregulated in the osteosarcoma tissues (Fig. 2C).

Next, we assessed the correlation of *PLK1* expression with the prognosis of osteosarcoma patients. Due to the low incidence of osteosarcoma, we chose to analyze the data derived from sarcoma (SARC) that largely shares the stromal origin and multiple key pathological characteristics with osteosarcoma. Similar with the result in osteosarcoma, *PLK1* was also upregulated in sarcoma (Fig. 2D). Moreover, Kaplan-Meier analysis indicated that higher *PLK1* expression is associated with poor prognosis, reflected in the overall survival (OS) and recurrence free survival (RFS) (Fig. 2E, F).

### *PLK1* is indispensable for the proliferation of osteosarcoma cells in vitro and the tumor growth of osteosarcoma xenograft in vivo

**Fig. 3:**
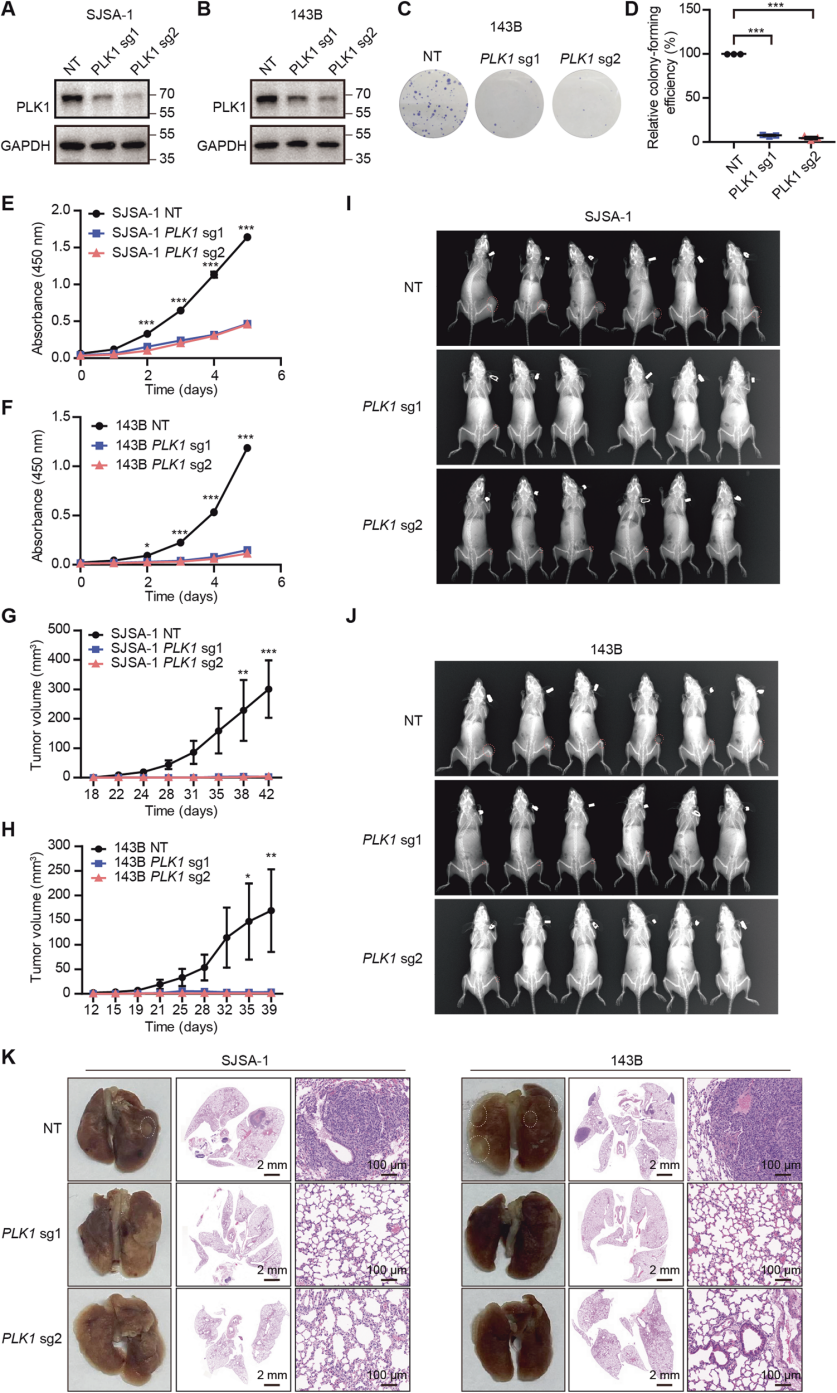
*PLK1* knockout effectively suppressed the development of OS in vitro and in vivo. Western blot analysis of SJSA-1 (**A**) and 143B (**B**) cells transduced with *PLK1* sgRNAs. **C**, **D** Colony formation ability was assessed in 143B cells transduced with *PLK1* sgRNAs. Values indicate mean ± SEM from three independent experiments. Cell viability was assessed in SJSA-1 (**E**) and 143B (**F**) cells transduced with *PLK1* sgRNAs. Values indicate mean ± SEM from three independent experiments. Tumor volumes of NT control group and *PLK1* sgRNA group (*n* = 6 mice of per group) from SJSA-1 (**G**) and 143B cells (**H**). Values indicate mean ± SEM. X-ray images of NT control group and *PLK1* sgRNA group from SJSA-1 (**I**) and 143B (**J**) cells in **G** and **H**. **K** Representative images and H&E of isolated lungs obtained in **G** and **H**. Unpaired and two-tailed t-tests were used to determine *P* values for **D** and two-way ANOVA with Bonferroni post-test were used for **E**–**H**. *p < 0.05; **p < 0.01; ***p < 0.001.

In order to exclude no-specific effects of inhibitor, we first investigated the role of *PLK1* in SJSA-1 and 143B osteosarcoma cells by deleting *PLK1* using two independent *PLK1* sgRNAs from the kinome-wide CRISPR-Cas9 screens (Fig. 3A, B). Cell growth and colony formation assays showed that *PLK1* KO significantly suppressed the proliferation of SJSA-1 and 143B cells in vitro in comparison with the non-targeting (NT) control (Fig. 3C–F). We also orthotopically inoculated the SJSA-1 and 143B cells stably expressing NT or *PLK1* sgRNAs into the tibial plateau of mice. *PLK1* KO led to a dramatic reduction in tumor size (Fig. 3G–J, Supplementary Fig. S4A, B) and a decrease in lung metastasis rate (Fig. 3K). More than 30% of mice inoculated the control cells experienced lung metastasis, which was completely constrained by the *PLK1* KO. No morphological changes in other major organs were observed (Supplementary Fig. S4C). Thus, *PLK1* is required for the development of OS.

### PLK1 inhibition effectively suppresses the proliferation and migration of osteosarcoma cells in vitro

**Fig. 4:**
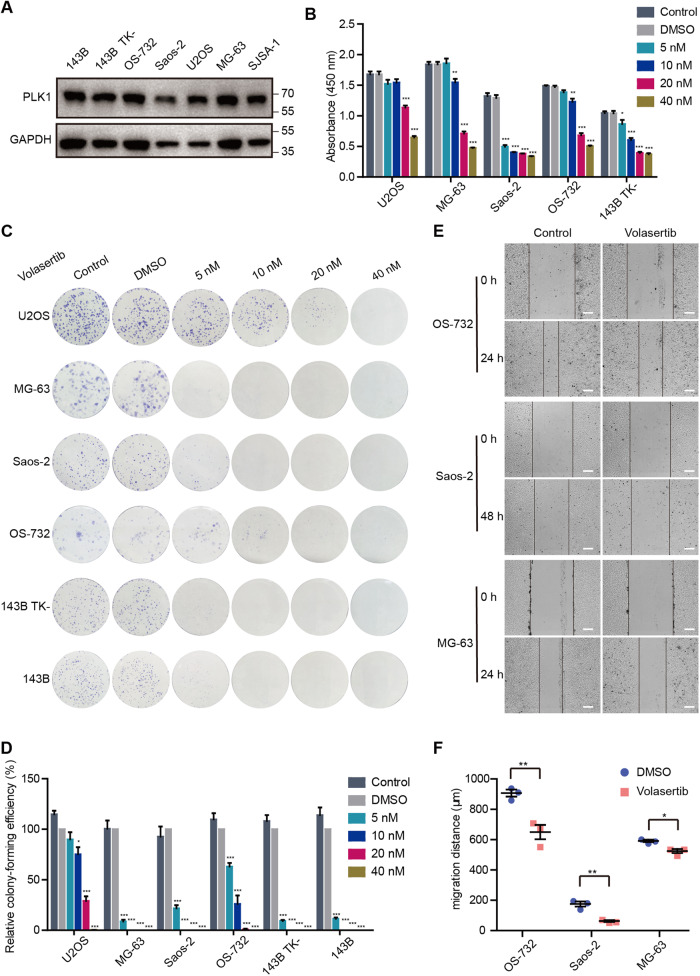
Multiple osteosarcoma cell lines are sensitive to PLK1 inhibitor Volasertib in vitro. **A** Western blot analysis of PLK1 protein levels of multiple osteosarcoma cells. Cell viability (**B**), colony formation ability (**C**, **D**) and cell migration (**E**, **F**) were assessed in multiple osteosarcoma cell lines treated with indicated doses of Volasertib. For cell migration assay, the doses in OS-732 and Saos-2 is 20 nM, and MG-63 is 10 nM. Scale bar, 300 µm. Values indicate mean ± SEM from at least three independent experiments and unpaired and two-tailed t-tests were used to determine *P* values. *p < 0.05; **p < 0.01; ***p < 0.001.

Volasertib is a highly potent pharmacological agent targeting PLK1 [17] and is currently in phase III clinical trials (http://www.clinicaltrials.gov). We further investigated the role of *PLK1* in osteosarcoma by inhibiting its activity in osteosarcoma cell lines with Volasertib. PLK1 is expressed in U2OS, MG-63, Saos-2, OS-732, 143B TK-, 143B and SJSA-1 cell lines tested, though the level of expression varies between cell lines (Fig. 4A). As shown in Fig. 4B, Volasertib (≥10 nM) significantly suppressed the growth of U2OS, MG-63, Saos-2, OS-732, 143B TK- cell lines. Similar inhibitory effects were observed when examining the colony formation of different cell lines exposed to the indicated doses of Volasertib in U2OS, MG-63, Saos-2, OS-732, 143B TK- and 143B cells (Fig. 4C, D). In addition, PLK1 inhibition by Volasertib led to significant reduction in migration in MG-63, Saos-2, and OS-732 cells (Fig. 4E, F). Interestingly, we noticed that the difference in Volasertib sensitivity was consistent in the impact on cell growth, colony formation, and migration. These findings further demonstrates that *PLK1* is a key regulator of these carcinogenic characteristics of osteosarcoma cells.

### PLK1 inhibition arrests the cell cycle at G2/M phase and triggers apoptosis

**Fig. 5:**
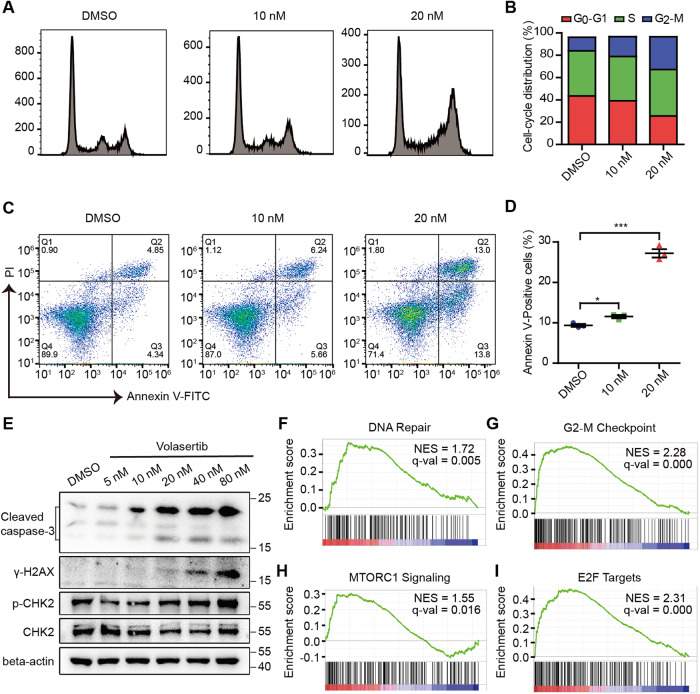
PLK1 inhibition enhances G2-M arrest and apoptosis. **A**, **B**, cell cycle distribution as determined by flow cytometry in Saos-2 cells treated with either DMSO or Volasertib (10 nM or 20 nM) for 72 h. **C** Representative Annexin V and PI staining FACS plots for assessing apoptosis in Saos-2 cells treated with either DMSO or Volasertib (10 nM or 20 nM) for 72 h. **D** Quantification of Annexin V positive cells (Q2 and Q3) in (**C**). **E** Western blot analysis of cleaved caspase-3, γ-H2A.X, p-CHK2, and CHK2 in Saos-2 cells treated with either DMSO or indicated doses of Volasertib for 72 h. GSEA analysis of RNA-seq data from Saos-2 cells treated with either DMSO or Volasertib (20 nM) showing significant upregulation of the DNA repair (**F**), G2-M checkpoint (**G**), MTORC1 signaling (**H**) and E2F targets (**I**) pathways. NES: normalized enrichment score. Values indicate mean ± SEM from three independent experiments and unpaired and two-tailed t-tests were used to determine *P* values. *p < 0.05; **p < 0.01; ***p < 0.001.

Next, we investigated the mechanisms underlying the inhibitory effect of Volasertib. The cell cycle of Saos-2 cells was altered by Volasertib in a dose-dependent manner (Fig. 5A, B). It was arrested at G2/M phase, which resulted in significant increase in the proportion of the G2-M population, in line with PLK1’s pivotal roles in cell cycle, such as activation of CDK1-cyclin B to promote G2-M transition as well as centrosome maturation separation during G2-M transition [18–20] (Fig. 5A, B). Cell apoptosis was also triggered by the PLK1 inhibition, reflected in the increase of the apoptotic population specifically stained by Annexin V (Fig. 5C, D). The cleavage of caspase-3 was specifically responsive with increased doses of Volasertib in parallel to the activation of the DNA damage response signaling pathway, indicated by the presence of γ-H2AX and the phosphorylation of CHK2, consistent with previous observations [21, 22] (Fig. 5E). Subsequently, RNA-seq was performed to identify genes and pathways affected by PLK1 inhibition. Gene Set Enrichment Analysis (GSEA) showed that DNA damage repair (DDR), G2-M checkpoint, MTORC1 signaling, and E2F targets pathways were significantly upregulated following PLK1 inhibition (Fig. 5F–I), suggesting a regulatory role by *PLK1* in DDR as well as in MTOR1-, and E2F1-mediated transcriptional programs [23–25].

Taken together, PLK1 inhibition by Volasertib-triggered DNA damage response, arrested the cell cycle at G2-M phase, and led to caspase-3-dependent cell apoptosis.

### Volasertib is potent in suppressing the tumor growth in the osteosarcoma PDX model

**Fig. 6:**
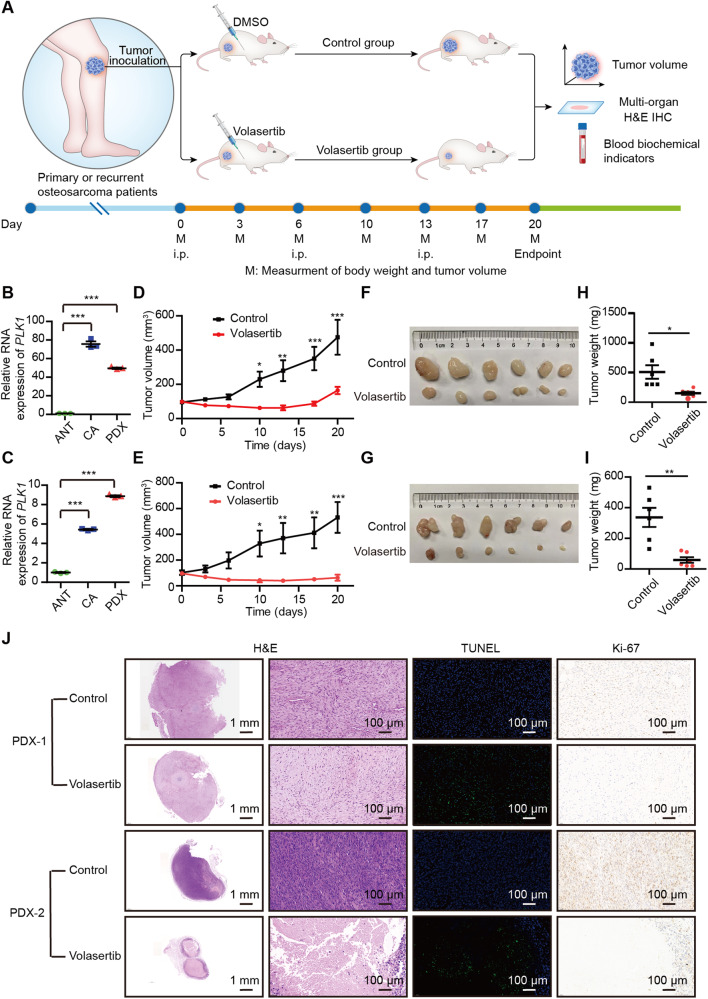
PLK1 inhibitor Volasertib potently inhibits growth of osteosarcoma PDXs. **A** Schematic workflow of the experiments. Relative *PLK1* expression assessed by qRT-PCR in PDX-1 (**B**) and PDX-2 lines (**C**) in comparison with the matched adjacent normal tissues (ANT) and tumor tissues (CA). Tumor volumes of vehicle control group and Volasertib group (*n* = 6 mice of per group) from PDX-1 (**D**) and PDX-2 lines (**E**). **F, G** Tumor images obtained in **D** and **E**. **H**, **I** Tumor weights obtained in **D** and **E**. **J** Representative H&E, TUNEL, Ki-67 staining of PDX tumors. Values indicate mean ± SEM. Unpaired and two-tailed *t*-tests were used to determine *P* values for **B**, **C**, **H**, and **I** and two-way ANOVA with Bonferroni post-test were used for **D** and **E**. *p < 0.05; **p < 0.01; ***p < 0.001.

To provide preclinical proof-of-concept therapeutic actionability of PLK1 inhibitor, we further examined the impact of PLK1 inhibition in osteosarcoma PDX models. Human osteosarcoma tissues were derived from two individual patients and were grafted to SCID Beige mice respectively to generate two independent lines of PDX model (hereafter PDX-1 and PDX-2) (Fig. 6A). *PLK1* was highly expressed in both PDX-1 and PDX-2 lines (Fig. 6B, C). Once xenografts were established, both lines were treated with either Volasertib (25 mg/kg) or vehicle as control once weekly for 20 days (Fig. 6A). In both PDX lines, PLK1 inhibition by Volasertib led to dramatic reduction in tumor size (Fig. 6D–G) and weight (Fig. 6H, I). To a large extent, growth inhibition in vivo as in vitro was mediated by Volasertib-triggered cell apoptosis, as demonstrated by higher TUNEL staining and lower Ki-67 staining in comparison with the vehicle control group, especially in the PDX-2 line derived from the patient with recurrent osteosarcoma amputation (Fig. 6J). While no significant histological alteration was observed in other organs, including lung, kidney, heart, liver, and spleen (Supplementary Fig. S5A). Neither in the level of the blood biochemical indicators such as total protein, albumin, total bilirubin, uric acid, urea, urea nitrogen, alanine aminotransferase (ALT), creatinine, and β2-microglobulin in blood (Supplementary Fig. S5B–J). Therefore, Volasertib exhibited great antitumor efficacy with minimal toxicity in our PDX models.

## Discussion

The current treatment of osteosarcoma is greatly challenged by the lack of available pharmaceutical targets. In this work, we systematically explored human kinome for the potential therapeutic candidates using comprehensive CRISPR-Cas9 knockout screens. Among a cohort of essential genes identified in human osteosarcoma cells, *PLK1*, a cell cycle master regulator, stood out as the top hit. Considering the great potential in its therapeutic value, we further investigated the role of *PLK1* in osteosarcoma using CRISPR knockout and a specific inhibitor Volasertib that has undergone multiple clinical trials.

Interestingly, *PLK1* as the top hit in our screens had been also identified in a parallel screen in 143B, MG-63, and U2OS-MTX300 osteosarcoma cells [26], thereby confirming the robustness of our identification as well as the important role of *PLK1* in osteosarcoma.

*PLK1* has been proposed as a therapeutic target for osteosarcoma [26–34]. With effective PDX models of primary and recurrent osteosarcoma patients, we provided further clinical-relevant evidence for *PLK1* targeted therapies and demonstrated the high specificity of Volasertib as a promising clinical candidate.

Furthermore, we also confirmed that *PLK1* dysregulation is closely associated with osteosarcoma tumorigenesis, particularly with poor prognosis, in line with the results from previous independent studies [35, 36]. However, it still remains largely undetermined for the specificity and effectiveness in targeting different tumor types [37, 38]. We show here that Volasertib-based therapy is effective and specific in inhibiting osteosarcoma growth both in vitro and in vivo, thus facilitating further development of PLK1 targeted therapies.

## Materials and methods

### Cell culture

U2OS, Saos-2, 143B TK-, and MG-63 cell lines were obtained from the National Infrastructure of Cell Line Resource (Beijing, China), and SJSA-1 and 143B cell lines were obtained from the American Type Culture Collection, which are authenticated by their short tandem repeat (STR) profiling, and OS-732 was preserved in our laboratory [39]. Saos-2 and U2OS cells were maintained in McCoy’s 5A medium (Corning), SJSA-1 and OS-732 cells were maintained in PRMI 1640 medium (Corning), and other lines in Dulbecco’ modification Eagle’ Medium (DMEM, Corning), supplemented with 10% FBS (Gibco) and 1% streptomycin-penicillin (Gibco). All cultures were maintained at 37 °C with 5% CO_2_.

### Small molecule inhibitors

Volasertib (S2235), Dinaciclib (S2768), Alisertib (S1133), and Barasertib (S1147) were purchased from Selleckchem.

### Lentivirus production of the human kinome-wide knockout library

HEK293T cells with low passage were plated at 1.6 × 10^7^/15 cm-dish on the day before transfection. The library plasmid and lentiviral helper plasmids were co-transfected into 80–90% confluent cells using Neofect DNA transfection reagent (Neofect, TF201201) at the following ratio, Human kinome CRISPR knockout library (Brunello, Addgene Cat# 1000000083): psPax2 (Addgene Cat# 12260, RRID: Addgene_12260): pMD2.G (Addgene Cat# 12259, RRID: Addgene_12259) = 7: 5: 2. 16~20 h post transfection, the culture media were replaced with viral production medium (Lonza). At 48 h post transfection, viral supernatant was collected, passed through 0.45 µm filters (Millipore SteriCup 250 mL; Millipore), and aliquoted for storage at −80 °C.

### Kinome-wide CRISPR-Cas9 knockout screens

U2OS, Saos-2, or OS-732 cells were transduced with lentivirus of kinome library at multiplicity of infection (MOI) of 0.3. For each cell line, two infection replicates were performed. Forty-eight hours post infection, transduced cells were selected with the puromycin (Thermo Fisher Scientific) for 48 h. 2 × 10^7^ transduced cells were collected as pre-screening cells (T0); the remaining cells were cultured for about 14 population doubling time and then collected as post-screening cells (Tn). The genomic DNA (gDNA) were extracted using Blood & Cell Culture DNA Maxi Kit (QIAGEN).

Library preparation for sequencing were accomplished by two rounds of PCR. For the first-round PCR, sgRNA flanking regions were amplified using Next High-Fidelity 2 × PCR Master Mix (New England Biolabs). The second-round PCR was performed to attach Illumina index and adapters using 2 µl of the first-round amplicons as templates for each reaction. The primers used in two rounds PCR are as follows:

First-round forward: 5′-AATGGACTATCATATGCTTACCGTAACTTGAAAGTATTTCG-3′;

First-round reverse: 5′-GTGACTGGAGTTCAGACGTGTGCTCTTCCGATCTACTGACGGGCACCGGAGCCAATTCC-3′;

Second-round forward: 5′-AATGATACGGCGACCACCGAGATCTACACTCTTTCCCTACACGACGCTCTTCCGATCTTCTTGTGGAAAGGACGAAACACCG-3′;

Second-round reverse: 5′-CAAGCAGAAGACGGCATACGAGATNNNNNNGTGACTGGAGTTCAGACGTG-3′ (NNNNNN stands for 6 bp index).

The second-round PCR products were gel-purified using TIANgel Midi Purification Kit (TIANGEN) and further processed with AMPure XP beads (Beckman Coulter). The diluted PCR libraries were sequenced on Hiseq X10 platform (Illumina).

### Analysis of CRISPR screen data

Fastx_barcode_splitter.pl command of FASTX-Toolkit software (version 0.0.14, http://hannonlab.cshl.edu/fastx_toolkit/index.html) was used to process the sequencing data. 20 bp of sgRNA sequences were extracted using FASTX-Toolkit software and then mapped to the kinome-wide library using bowtie (version 1.2.2) [40, 41] with permission of single nucleotide mismatch. The sgRNA counts were quantified using python. Gene essentiality scores (beta scores) were defined using maximum-likelihood estimation (MLE) module of Model-based analysis of genome-wide CRISPR/Cas9 knockout (MAGeCK) software (version 0.5.7) [16].

### qRT-PCR

Total RNA was harvested from cells or tissues using TRIzol (Ambion) and 2 μg of total RNA were reversely transcribed into cDNA using the RevertAid First Strand cDNA Synthesis Kit (Thermo Fisher Scientific). Quantitative reverse transcription PCR (qRT-PCR) was performed using LightCycler 480 SYBR Green I Master reaction mix (Roche). The primers sequence used for qRT-PCR were the following.

*PLK1* forward: 5′-CAGGCAAGAGGAGGCTGAGGAT-3′;

*PLK1* reverse: 5′-TGTAGAGGATGAGGCGTGTTGAGT-3′;

*GAPDH* forward: 5′-TGAAGGTCGGAGTCAACGG-3′;

*GAPDH* reverse: 5′-TCCTGGAAGATGGTGATGGG-3′;

### Western blot

Cells or tissue samples were lysed with RIPA lysis buffer (Beyotime Biotechnology) supplemented with phosphatase (PhosSTOP, Roche) and protease (cOmlpete, Roche) inhibitor cocktails. Immunoblotting was performed as described previously [42]. The following antibodies were used: anti-PLK1 (Cell Signaling Technology, Cat# 4513, RRID: AB_2167409); anti-Cleaved Caspase-3 (Cell Signaling Technology, Cat# 9661, RRID: AB_2341188); anti-phospho-Histone H2A.X (Millipore, Cat# 05-636, RRID: AB_309864); anti-phospho-CHK2 (Cell Signaling Technology, Cat# 2197, RRID: AB_2080501); anti-CHK2 (Cell Signaling Technology, Cat# 6334, RRID: AB_11178526); anti-GAPDH (Zhongshanjinqiao Biotechnology, Cat# TA-08, RRID: AB_2747414) and anti-beta-actin (Cell Signaling Technology, Cat# 4970, RRID: AB_2223172).

### Analysis of public database

TNMplot web tool (https://tnmplot.com) was used to determine *PLK1* expression in the samples derived from TARGET database [43]. UCSC Xena web tool (https://xena.ucsc.edu/) was used to analyze the *PLK1* expression in the samples derived from TCGA database [44]. The differential expression of *PLK1* associated overall survival (OS) and recurrence free survival (RFS) were analyzed by Kaplan-Meier Plotter (https://kmplot.com) [45].

### Generation of *PLK1* knockout cells

Non-targeting (NT) and two *PLK1* sgRNAs were cloned into lentiCRISPR v2 vector (Addgene Cat#52961, RRID: Addgene_52961) and transfected into HEK293T cells for lentivirus production. SJSA-1 and 143B cells were transduced with lentivirus containing NT and *PLK1* sgRNAs for 24 h, and puromycin was added to select the transduced cells. The sgRNA sequences were the following.

NT sgRNA: 5′-CTGAAGGTGTCTGGCAGAGC-3′;

*PLK1* sg1: 5′-CCTGCCTGACCATTCCACCA-3′;

*PLK1* sg2: 5′-ACCGGCGAAAGAGATCCCGG-3′;

### Cell viability assay

SJSA-1 (1200 cells per well) and 143B cells (1500 cells per well) transduced with *PLK1* sgRNAs or the NT sgRNA were plated in 96-well plates and cell viability was measured for 5 days. U2OS, Saos-2, OS-732, 143B TK-, or MG-63 cells (1000 cells per well) were plated in 96-well plates, incubated overnight, and treated with Volasertib for 4 days. Cell viability was determined by measuring the absorbance at 450 nm with Cell Counting Kit-8 (DOJINDO).

### Clonogenic survival assay

The clonogenic survival assay was performed as described previously [46]. Briefly, 143B cells (800 cells per well) transduced with *PLK1* sgRNAs were plated in triplicates on a 6-well plate, cultured for 8 days, fixed with formalin, and stained with 0.1% crystal violet. U2OS, Saos-2, OS-732, 143B TK-, MG-63, or 143B cells (600 cells per well) were seeded in triplicates on a 6-well plate. After 24 h of incubation, cells were treated with the indicated doses of Volasertib for 4–13 days, fixed with formalin, and stained with 0.1% crystal violet. Colonies containing more than 50 cells were calculated.

### Cell migration assay

Saos-2, OS-732, or MG-63 cells were seeded in 6-well plate (5 × 10^5^/well). Once the cells reached 90% confluence, scratches were generated in the center of each well using a 200-μl tip. Cells were then treated with DMSO or Volasertib (20 nM for OS-732 and Saos-2 cells, and 10 nM for MG-63 cells) for 24 or 48 h. The wounds were photographed to assess cell migration.

### Cell-cycle analysis

Cell-cycle assays were performed using Cell Cycle Detection Kit (KGA511, KeyGEN BioTECH). In brief, cells were harvested by trypsinization, fixed in ice-cold 70% ethanol at 4 °C overnight, stained with propidium iodide for 30 min at room temperature in the dark, and analyzed by Cytoflex instrument (Beckman, IN, USA). The data were analyzed using FlowJo software (version 10.8.1).

### Apoptosis analysis

Cells were harvested by trypsinization and stained with FITC-labeled Annexin V and propidium iodide for 15 min at room temperature in the dark (BD Biosciences). The apoptotic cells were detected with Cytoflex instrument (Beckman). FlowJo software (version 10.8.1) was used for processing the data.

### RNA-seq and data analysis

Total RNA was extracted using RNAprep Pure Micro Kit (TIANGEN) according to the manufacturer’s instructions. Sequencing was performed on a Novaseq platform. Paired-end RNA-seq reads were aligned to the human transcriptome genome (Grch37) using HISAT2 software (version 2.1.0) [47]. StringTie software (version 1.3.4) [48] was used to assemble transcripts and estimate gene abundances. Gene Set Enrichment Analysis (GSEA) were performed using the GenePattern platform of GESA website (https://www.genepattern.org/) [49].

### Patient samples

Osteosarcoma and matched adjacent normal tissues from surgery or biopsy were obtained from Beijing Jishuitan Hospital. A total of 20 paired osteosarcoma specimens were collected, including 18 primary osteosarcoma and matched adjacent muscle tissues, 1 lung metastatic specimen and matched adjacent lung tissues, and 1 breast metastatic specimen and matched adjacent breast tissues.

This study was approved by the Ethics Committee of Beijing Jishuitan Hospital (Grant No. K2022-117-00). Written informed consent was obtained from all patients.

### Cell-derived xenografts (CDX) assay and patient-derived xenografts (PDXs) assay

All animal procedures were performed in accordance with the Guidelines for Care and Use of Laboratory Animals of Beijing Jishuitan Hospital and approved by the Animal Ethics Committee of the Beijing Jishuitan Hospital (202104-01).

For CDXs assay, SJSA-1 and 143B cells transduced with lentivirus of NT or *PLK1* sgRNAs were inoculated into the bone marrow cavity of 6- to 8-week-old female BALB/c nude mice (6 mice/each group) through the right tibial plateau. Body weights and tumor volume were measured every 3~4 days. Tumor volumes were evaluated by caliper measurements using the following formula: (length × width^2^)/2. After about 40 days of inoculation, the mice were euthanized, photographed by X-ray and lumps were removed.

For PDXs assay, fresh tumor tissues collected from two osteosarcoma patients were minced into small pieces and subcutaneously inoculated into 6- to 8-week-old female SCID Beige mice to generate PDXs models. The grafts were closely monitored. Once the PDXs were palpable, mice bearing PDXs were randomly divided into two subgroups and administered intraperitoneally with either Volasertib (25 mg/kg body weight) or vehicle control once a week for 20 days. For the duration of the experiments, tumor volumes and the body weight of mice were measured every 2~3 days. At the end of the experiment, all mice were sacrificed, lumps were removed, weighed, and photographed.

### Hematoxylin-eosin (HE) staining, immunohistochemistry (IHC) and apoptotic assay

Tumor tissues and visceral organs from mice were fixed with 10% neutral formalin and embedded in paraffin. After deparaffinization and rehydration, tissues were stained with hematoxylin and eosin, dehydrated in a gradient series of ethanol and cleared in xylene. For IHC, tumor tissues on slides were dewaxed, hydrated and incubated with antigen retrieval solution. The tissues on slides were then incubated overnight with primary antibody against Ki-67 (Abcam, Cat# ab15580, RRID: AB_443209) at 4 °C. Following intensive washes, the tissues on slides were incubated with goat anti-rabbit IgG secondary antibody followed by chromogenic reaction with Diaminobenzidine (DAB). Terminal deoxynucleotidyl transferase dUTP nick-end labeling (TUNEL) assay for assessing apoptotic status in tumor tissues were conducted by using Cell Death Detection Kit (GPB1829, Genepool) according to the manufacturer’s instructions. Images were captured by Pannoramic MIDI (3D HISTECH, Budapest, Hungary).

### Statistical analysis

GraphPad Prism 5 were used for statistical analysis. Statistical significance between groups were calculated using two-tailed unpaired Student *t*-test or two-way ANOVA with Bonferroni post-test. *p < 0.05; **p < 0.01; ***p < 0.001.

## Supplementary information

Supplementary material

Full and uncropped western blots

Supplementary Table 1

Reproducibility Checklist

## Data Availability

The datasets generated in this study are available on the NCBI BioProject Archive under the accession no. PRJNA970863.
