# Supplementary material for: Kinome-wide CRISPR-Cas9 knockout screens revealed PLK1 as a therapeutic target for osteosarcoma
Source: Cell Death Discov. 2023 Jul 7;9:231. doi: 10.1038/s41420-023-01526-7 (PMC10328921; doi:10.1038/s41420-023-01526-7)
Supplement: Supplementary file 1 — Supplementary material [file 41420_2023_1526_MOESM1_ESM.pdf]

Supplementary material

Supplementary Table legends

Table S1 - Table S1 lists all the candidate genes identified from the kinome-wide CRISPR-Cas9 screens.

Supplementary Figures and Figure legends

Figure S1

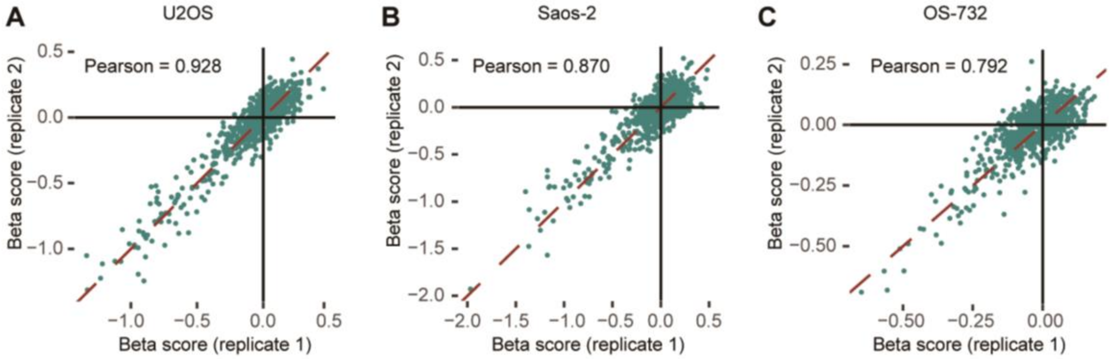

Figure S1. Correlation of sgRNA beta scores calculated from two infection replicates of U2OS (A), Saos-2 (B) and OS-732 (C) cells. Pearson correlation coefficients were used.

Figure S2

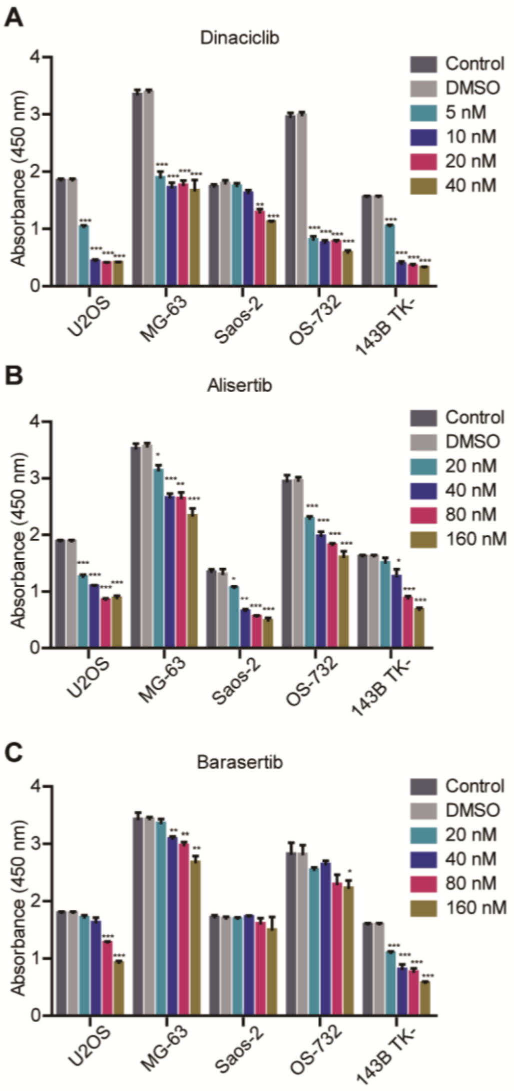

Figure S2. Validation of candidate essential genes by using specific chemical inhibitors. A-**C**, Cell viabilities of multiple osteosarcoma cell lines treated with indicated doses of Dinaciclib (**A**), Alisertib (**B**), and Barasertib (**C**). Values indicate mean ± SEM from three independent experiments and unpaired and two-tailed t-tests were used to determine *P* values. *, $p < 0.05$; **, $p < 0.01$; ***, $p < 0.001$.

Figure S3

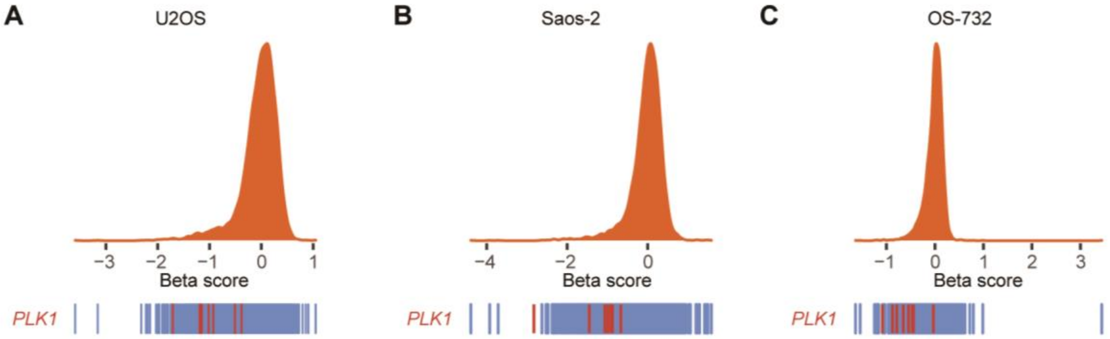

Figure S3. Frequency histograms of sgRNA beta scores showing essentiality of *PLK1* in U2OS (A), Saos-2 (B) and OS-732 (C) cells. The beta scores of individual sgRNAs targeting *PLK1* gene are marked by red lines.

Figure S4

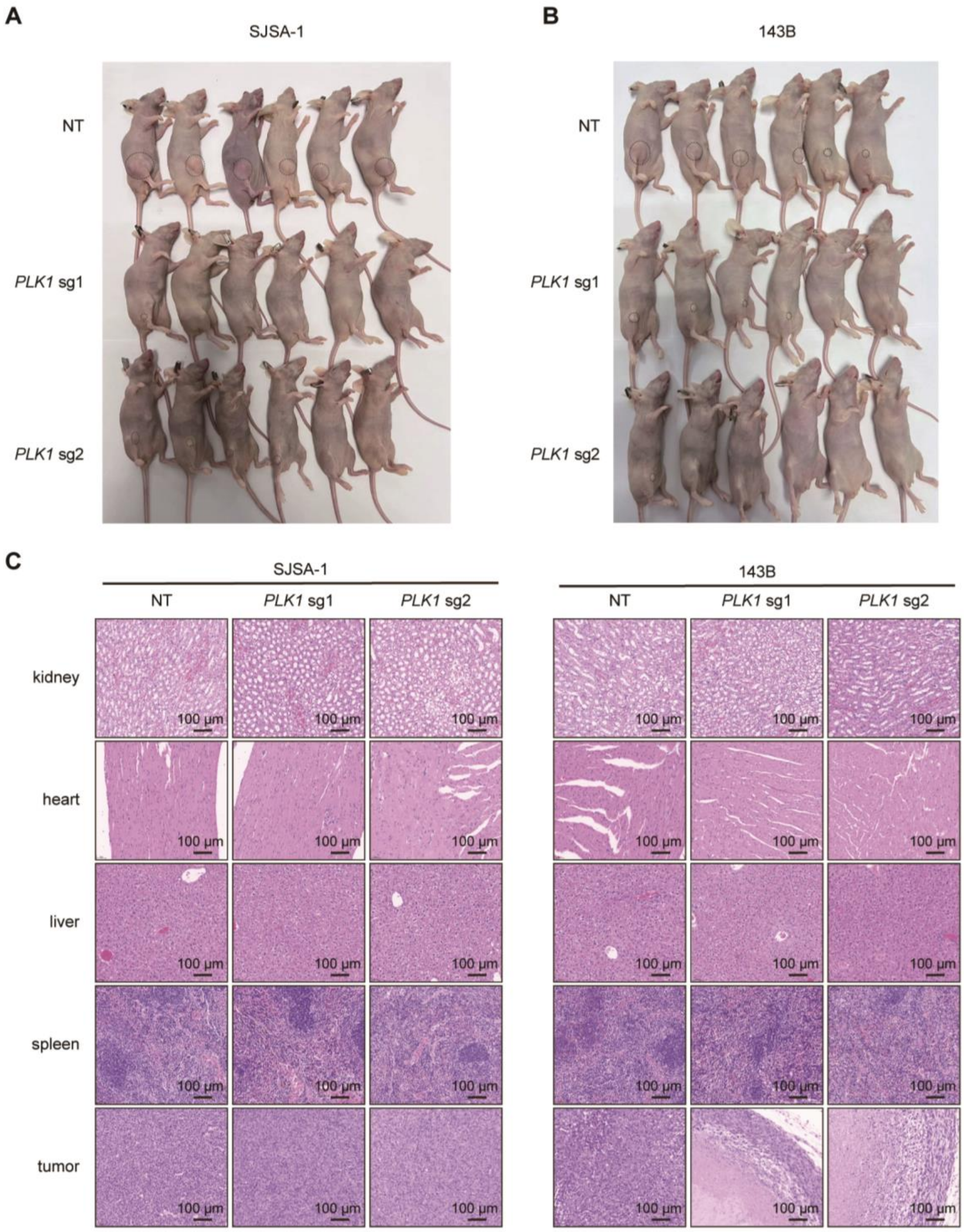

Figure S4. *In vivo* effects of *PLK1* knockout on mice bearing CDX. A and **B**, Images of NT control group and *PLK1* sgRNA group from SJSA-1 (**A**) and 143B (**B**) cells. **C**, Representative H&E staining of kidney, heart, liver, spleen and tumor of mice from each group.

Figure S5

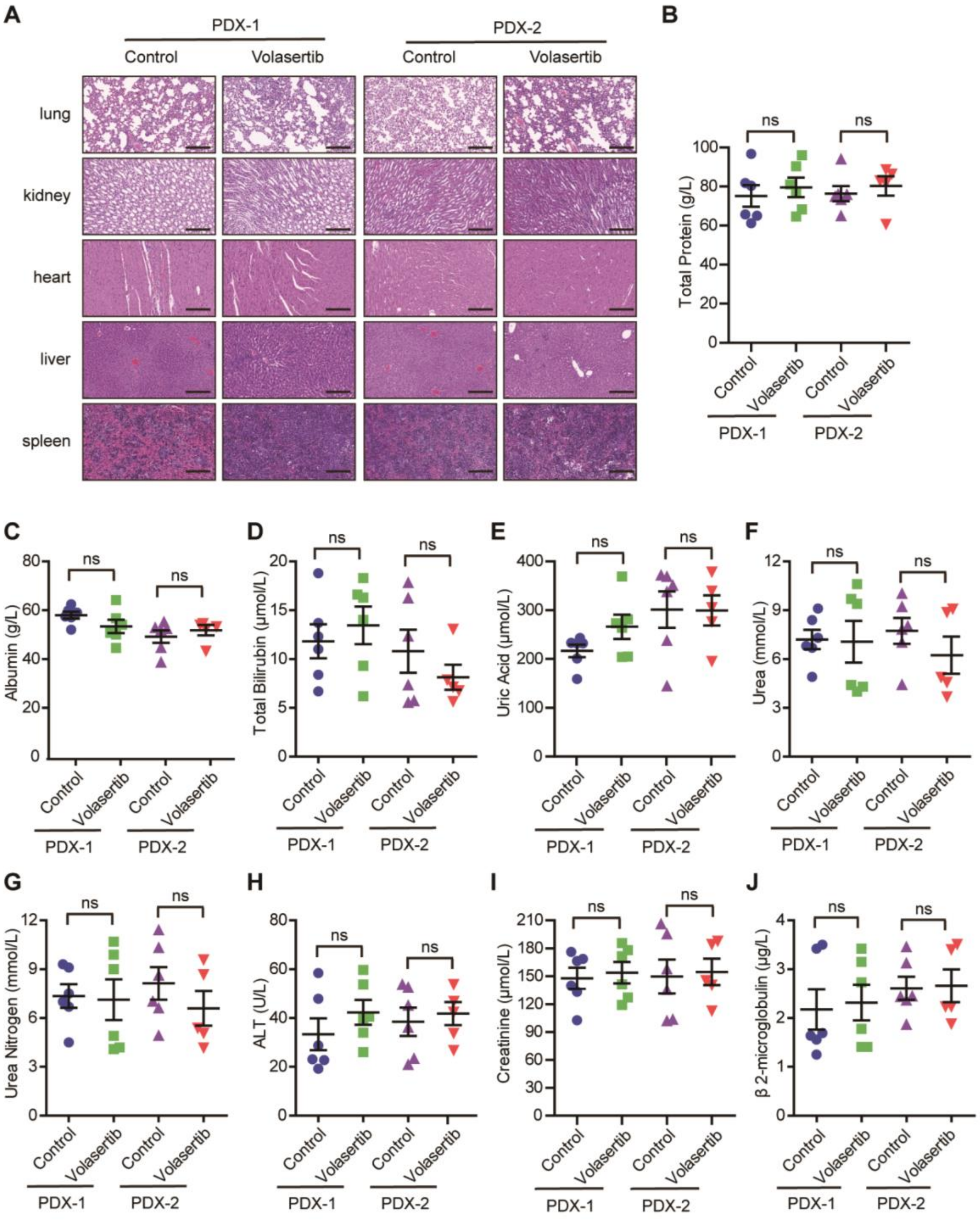

Figure S5. *In vivo* effects of PLK1 inhibitor Volasertib on mice bearing PDX. A, Representative H&E staining of lung, kidney, heart, liver, and spleen of mice from each group. Scale bar, 200 μm. **B**-**J**, Concentration of total protein (**B**), albumin (**C**), total bilirubin (**D**), uric acid (**E**), urea (**F**), urea nitrogen (**G**), alanine aminotransferase ALT (**H**), creatinine

(**I**), and β2-microglobulin (**J**) in blood of mice from each group. Values indicate mean ± SEM and unpaired and two-tailed t-tests were used to determine *P* values. *, $p < 0.05$; **, $p < 0.01$; ***, $p < 0.001$.
