# Supplementary material for: Kinome-wide CRISPR-Cas9 knockout screens revealed PLK1 as a therapeutic target for osteosarcoma
Source: Cell Death Discov. 2023 Jul 7;9:231. doi: 10.1038/s41420-023-01526-7 (PMC10328921; doi:10.1038/s41420-023-01526-7)

Original image

Original image: Figure 2C

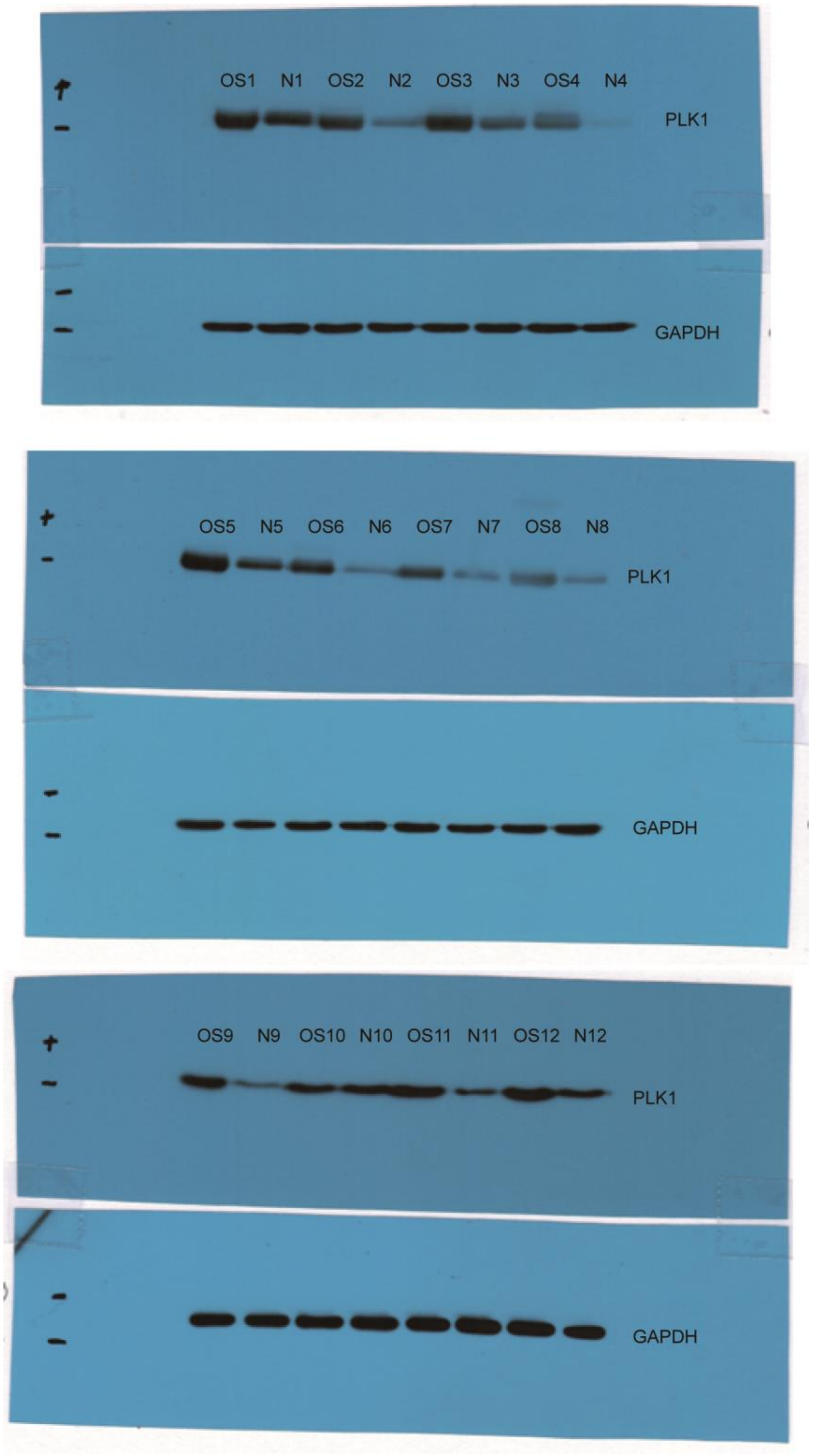

Original image: Figure 3A

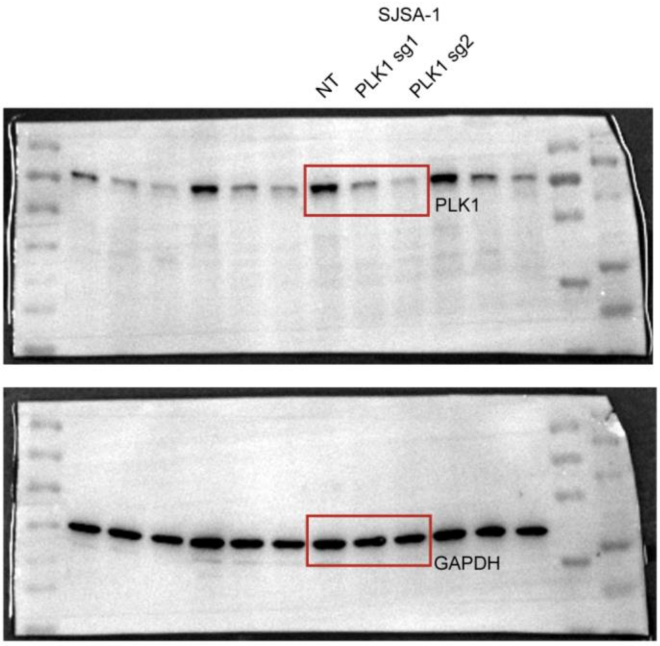

Original image: Figure 3B

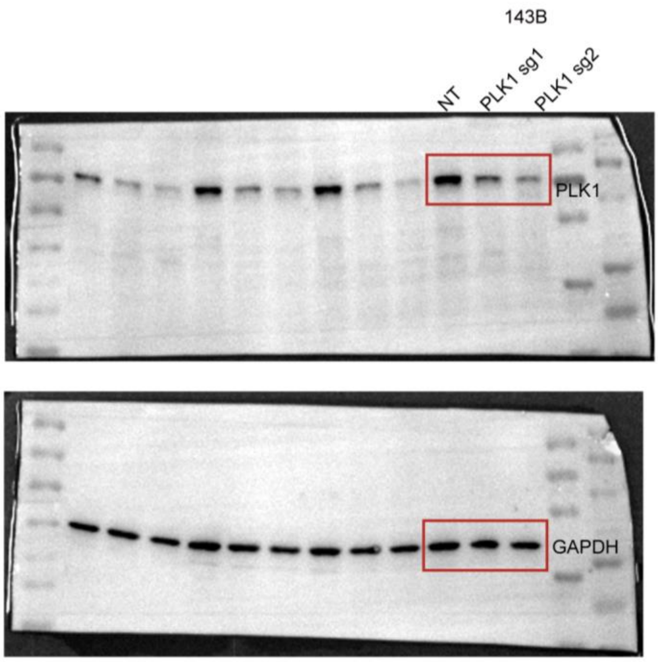

Original image: Figure 4A

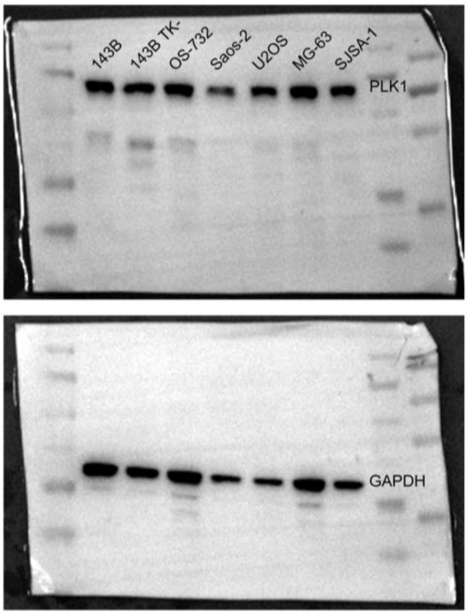

Original image: Figure 5E

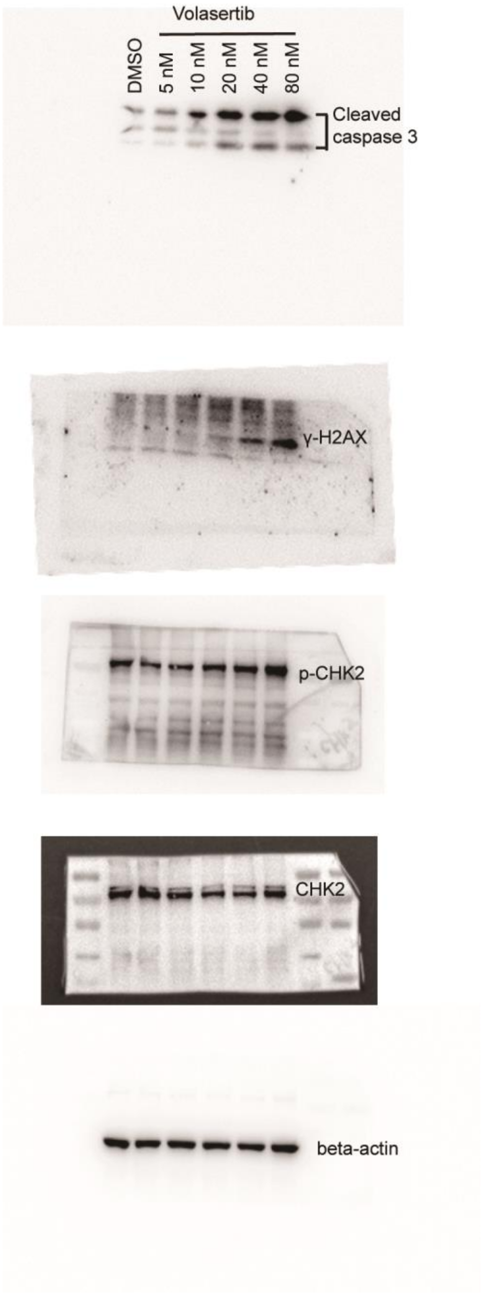

Supplement: Supplementary file 2 — Full and uncropped western blots [file 41420_2023_1526_MOESM2_ESM.pdf]
